# Electrophysiological Mechanisms of Brugada Syndrome: Insights from Pre-clinical and Clinical Studies

**DOI:** 10.3389/fphys.2016.00467

**Published:** 2016-10-18

**Authors:** Gary Tse, Tong Liu, Ka H. C. Li, Victoria Laxton, Yin W. F. Chan, Wendy Keung, Ronald A. Li, Bryan P. Yan

**Affiliations:** ^1^Department of Medicine and Therapeutics, Chinese University of Hong KongHong Kong, Hong Kong; ^2^Li Ka Shing Institute of Health Sciences, Chinese University of Hong KongHong Kong, Hong Kong; ^3^Tianjin Key Laboratory of Ionic-Molecular Function of Cardiovascular Disease, Department of Cardiology, Tianjin Institute of Cardiology, Second Hospital of Tianjin Medical UniversityTianjin, China; ^4^Faculty of Medicine, Newcastle UniversityNewcastle, UK; ^5^Intensive Care Department, Royal Brompton and Harefield NHS TrustLondon, UK; ^6^School of Biological Sciences, University of CambridgeCambridge, UK; ^7^Stem Cell and Regenerative Medicine Consortium, Li Ka Shing Faculty of Medicine, The University of Hong KongPokfulam, Hong Kong; ^8^Ming Wai Lau Centre for Reparative Medicine, Karolinska InstitutetSolna, Sweden; ^9^Department of Epidemiology and Preventive Medicine, Monash UniversityMelbourne, VIC, Australia

**Keywords:** arrhythmia, Brugada syndrome, sodium channel, repolarization, depolarization, risk stratification

## Abstract

Brugada syndrome (BrS), is a primary electrical disorder predisposing affected individuals to sudden cardiac death via the development of ventricular tachycardia and fibrillation (VT/VF). Originally, BrS was linked to mutations in the *SCN5A*, which encodes for the cardiac Na^+^ channel. To date, variants in 19 genes have been implicated in this condition, with 11, 5, 3, and 1 genes affecting the Na^+^, K^+^, Ca^2+^, and funny currents, respectively. Diagnosis of BrS is based on ECG criteria of coved- or saddle-shaped ST segment elevation and/or T-wave inversion with or without drug challenge. Three hypotheses based on abnormal depolarization, abnormal repolarization, and current-load-mismatch have been put forward to explain the electrophysiological mechanisms responsible for BrS. Evidence from computational modeling, pre-clinical, and clinical studies illustrates that molecular abnormalities found in BrS lead to alterations in excitation wavelength (λ), which ultimately elevates arrhythmic risk. A major challenge for clinicians in managing this condition is the difficulty in predicting the subset of patients who will suffer from life-threatening VT/VF. Several repolarization risk markers have been used thus far, but these neglect the contributions of conduction abnormalities in the form of slowing and dispersion. Indices incorporating both repolarization and conduction and based on the concept of λ have recently been proposed. These may have better predictive values than the existing markers.

## Introduction

The syndrome of right bundle branch block with ST segment elevation was first described by the Italian physicians, Drs Nava, Martini and Thiene, in 1989 (Martini et al., 1989). The term Brugada syndrome (BrS) was later introduced to describe a hereditary condition involving idiopathic ventricular tachycardia, ventricular fibrillation (VT/VF) and sudden cardiac death (SCD) in structurally normal hearts (Brugada and Brugada, 1992). Traditionally, BrS has been linked to loss-of-function mutations in the SCN5A gene, which encodes for the cardiac Na^+^ channel. Although SCN5A is the commonest affected gene, its mutations are only implicated in approximately a fifth of patients. In some pedigrees, affected members did not have mutations in this gene (Probst et al., 2009). Over the past decades, variants in 19 genes that affect the Na^+^, K^+^, Ca^2+^, and funny currents have been implicated. However, caution must be taken when interpreting such genetic variants, as these do not always contribute significantly to the BrS phenotype (Le Scouarnec et al., 2015). Other than BrS, Scn5a mutations have been associated with sick sinus syndrome (SSS; Benson et al., 2003), progressive cardiac conduction defect (PCCD, or Lenègre disease; Schott et al., 1999; Tan et al., 2001; Probst et al., 2003) and idiopathic VF without BrS findings (Akai et al., 2000). They can also lead to overlap disorders (Remme et al., 2008). For example, the p.Y1449C mutation was associated with conduction disease, Brugada syndrome and atrial flutter (Hothi et al., 2015). The differing disease phenotypes can partly be explained by the biophysical effects of the mutated *Scn5a* gene product (Liu et al., 2014). The same mutation can affect members of different families, or even members of the same family, differently, suggesting that other factors modify the behavior of the sodium channels.

BrS was initially estimated to account for 12% of the cases of SCD in the general population (Brugada and Brugada, 1992). However, recent epidemiological studies have demonstrated that its prevalence is much lower. Thus, in Southeast Asians, who are more predisposed to BrS than other ethnicities, only 0.1% showed a Brugada pattern (Ng et al., 2012). In Chinese subjects, the overall prevalence of BrS pattern was 3.3%, with 0.08% due to Type 1 BrS, and the remaining contributions from Types 2 and 3 (Juang et al., 2015). In Denmark, a low prevalence of BrS was found, at 0.001% (Holst et al., 2012). BrS has a male preponderance, affecting men four times more frequently than women and also affecting younger adults than infants or children (Nademanee et al., 1997). BrS can present with SCD (aborted or otherwise), syncope, palpitations or agonal breathing, leading them to undergo further investigation. Precipitating factors include increased vagal tone, fever, and other drugs such as tricyclic antidepressants and alcohol (Madeira et al., 2015; Achaiah and Andrews, 2016).

Aside from VT, BrS is associated with pre-excitation syndromes such as Wolff-Parkinson-White syndrome (Eckardt et al., 2001), and supraventricular tachycardia such as atrioventricular nodal reentrant tachycardia, atrial flutter or atrial fibrillation (Bordachar et al., 2004). Sinus node dysfunction in the form of prolonged sinoatrial node recovery time (Morita et al., 2004) has been observed in BrS. Conduction abnormalities such as reduced conduction velocity (CV) from the sinoatrial node to the atria or through the atria, and blocks such as atrial standstill are also observed (Takehara et al., 2004; Tse et al., 2016b).

Diagnosis of BrS is based on a Type 1 electrocardiographic (ECG) pattern of a coved-shaped ST segment elevation (STE) ≥2 mm and negative T-wave in the right precordial leads with or without drug challenge using a class I anti-arrhythmic agent such as flecainide (Priori et al., 2013). BrS can also be diagnosed when a type 2 (saddleback morphology, defined as a J-wave amplitude ≥2 mm but STE ≥1 mm) or type 3 (STE <1 mm with either a coved or saddleback morphology) ECG pattern is converted to type 1 by drug challenge. Clinical findings, such as agonal respiration during sleep, history of ventricular tachycardia or fibrillation (VT/VF), inducible VT/VF observed during an electrophysiological study (EPS) and family history of SCD or Type 1 (coved-type) ECG were part of the original diagnostic criteria, but have now been excluded in the latest consensus criteria (Priori et al., 2013). Some investigators have emphasized that having a Brugada pattern on the ECG should not automatically equate to a clinical diagnosis of BrS, especially in asymptomatic individuals without clinical signs or symptoms (Martini, 2016). Indeed, there are concerns that over-diagnosis may occur if this is based only on ECG criteria (Viskin et al., 2015). However, others pointed out that those with a Type 1 BrS ECG, even if only it is drug-induced, have a low but nevertheless elevated risk of sudden death compared to the general population and therefore patients should be aware of it (Andorin and Probst, 2016). A recent consensus conference report put forward the Shanghai BrS Score, where patients receive points for (i) ECG, (ii) clinical findings, (iii) family history and (iv) genetic results, and are stratified into non-diagnostic, possible or probable/definite BrS (Antzelevitch et al., 2016). The standard placement of ECG leads in the fourth intercostal spaces may not be sufficiently sensitive to detect the presence of BrS ECG patterns, as only mild ST segment elevation was observed (Shimizu et al., 2000). By contrast, the ST segment elevation is more pronounced when the leads are placed in the second intercostal spaces, in keeping with the arrhythmia origins in the right ventricular outflow tract (Curcio et al., 2016; Kumar and Kalman, 2016).

Traditionally, BrS was thought to be a Mendelian disease, having an autosomal dominant inheritance with incomplete penetrance (Sicouri et al., 2012), but this has been refuted (Gourraud et al., 2016). The genotype-phenotype correlation is poor; a recent study examined co-segregation of SCN5A mutations amongst large genotyped families, demonstrating that some affected family members did not carry the familial mutation (Probst et al., 2009). This suggests other mutations in other genes are responsible for BrS (Marian, 2009; Roden, 2010). Moreover, some putatively pathogenic genetic variants do not manifest clinically as abnormal phenotype (Van Driest et al., 2016). Therefore, caution must be taken in notifying patients of these incidental findings, which may not be clinically significant.

## Differential diagnosis: J-wave syndromes and other causes of Brugada pattern

J-wave syndrome is a term encompassing both BrS and early repolarization syndrome (ERS; Shinde et al., 2007; Antzelevitch et al., 2011; Wang et al., 2011). The letter “J” refers to the junctional point between the QRS and ST segment, representing the intersection between end of ventricular depolarization and onset of ventricular repolarization. Early repolarization was first described in 1951 (Grant et al., 1951). It has been defined as prominent or elevated J-point with notching or slurring of distal part of R wave in at least two contiguous leads (Mehta et al., 1999; Klatsky et al., 2003). Although traditionally seen as a benign finding (Mehta and Jain, 1995), early repolarization has subsequently been associated with a higher risk of SCD (Haïssaguerre et al., 2008; Rosso et al., 2008, 2012). The estimated prevalence of ERS is around 1 to 13% of the general population and associated with 15 to 70% of idiopathic VF cases (Haïssaguerre et al., 2008; Derval et al., 2011; Haruta et al., 2011). ERS has been reported to have an autosomal dominant origin with incomplete penetrance (Noseworthy et al., 2011; Nunn et al., 2011; Reinhard et al., 2011).

A group of heterogeneous conditions induce a Brugada ECG pattern, including “metabolism conditions, mechanical compression, myocardial ischaemia, pulmonary embolism, myocardial, and pericardial disease, ECG modulations and miscellaneous conditions” (Dendramis, 2016; Enriquez et al., 2016; Gottschalk et al., 2016). These must be distinguished from true BrS as these are potential reversible causes and do not necessitate invasive treatments such as implantable cardioverter-defibrillator (ICD) insertion.

## Electrophysiological mechanisms underlying arrhythmogenesis in Brugada syndrome

The Na^+^ channel consists of one α subunit (SCN5A) and one or two β subunits (SCN1B, SCN2B, SCN3B). Loss-of-function mutations in Scn5a have been associated with BrS (Chen et al., 1998), sick sinus syndrome (SSS; Benson et al., 2003), progressive cardiac conduction defect (PCCD, or Lenègre disease; Schott et al., 1999; Tan et al., 2001; Probst et al., 2003) and overlap disorders between these conditions (Remme et al., 2008). By contrast, gain-of-function Scn5a mutations are observed in long QT syndrome type 3 (Wang et al., 1995). BrS and LQTS share many similarities, existing in congenital or acquired forms (Havakuk and Viskin, 2016).

BrS has been associated with reduced *I*_Na_ and loss-of-function mutations in Scn5a. The latter can lead to impaired trafficking to the cell membrane, reduced expression and expression of non-functional proteins (Bezzina et al., 1999; Dumaine et al., 1999; Akai et al., 2000; Kyndt et al., 2001; Valdivia et al., 2004; Amin et al., 2005). Gating of Na^+^ channels can also be altered such as delayed activation, premature inactivation, enhanced slow inactivation and slower recovery from inactivation (Akai et al., 2000; Amin et al., 2005). Although the commonest mutations of BrS have been localized to SCN5a, accounting for 25% of cases (Probst et al., 2010), other genes have also been implicated. These include the different Na^+^ channel subunits, e.g., SCN5A, SCN1B, SCN2B, or SCN3B (Watanabe et al., 2008; Hu et al., 2009; Riuró et al., 2013; Ricci et al., 2014). Recently, SCN10A, a neuronal sodium channel gene, was proposed to be a putative causative gene in a large fraction of BrS cases (Hu et al., 2014a). Reduced *I*_Na_ can also arise from mutations in genes encoding for glycerol-3-phosphate dehydrogenase 1-like (GPD1-L) protein (London et al., 2007), MOG1 (Kattygnarath et al., 2011), sarcolemmal membrane-associated protein (SLMAP) (Ishikawa et al., 2012), the desmosomal component plakophilin-2 (Cerrone et al., 2014), FGF2 (fibroblast growth factor homologous factor-1) (Hennessey et al., 2013) and the transcriptional factor HEY2 (Bezzina et al., 2013; Boukens et al., 2013).

BrS can involve reduced *I*_Ca_ (Antzelevitch et al., 2007). LTCC consists of 4 protein subunits α1 (CACNA1C), β2 (CACNB2), α2 (CACNA2D), and δ (CACNA2D). Loss-of-function mutations in these genes can lead to abnormal trafficking, reduced expression or function of the LTCC, similar to the Na^+^ channel mutations (Antzelevitch et al., 2007; Burashnikov et al., 2010). However, a difference is that BrS secondary to loss-of-function of LTCCs are associated with shortened QT intervals, as opposed to classical BrS in which the QT interval is not normally altered. BrS has also been associated with gain-of-function mutations in genes encoding for proteins that constitute or modulate the different K^+^ channels. These include KCNE3, KCND3, and SEMA3A (semaphorin, an endogenous K^+^ channel inhibitor) responsible for *I*_to_ (Delpón et al., 2008; Giudicessi et al., 2011, 2012; Ohno et al., 2011; Nakajima et al., 2012; Boczek et al., 2014), KCNJ8 and ABCC9 (encoding for SUR2A, the ATP-binding cassette transporter for the K_ATP_ channel) determining *I*_K, ATP_ (Medeiros-Domingo et al., 2010; Hu et al., 2014b) and KCNH2 encoding for *I*_Kr_ (Wang et al., 2014). Most recently, dysfunction in the KCNAB2, which encodes the voltage-gated K^+^ channel β2-subunit, was associated with increased *I*_to_ activity and identified as a putative gene involved in BrS (Portero et al., 2016). Finally, loss-of-function mutations in HCN4 leading to decreased *I*_f_ and gain- or loss-of-function mutations in the transient receptor potential melastatin protein 4 gene (TRPM4) have also been implicated in BrS (Ueda et al., 2009; Liu et al., 2013).

**Figure 1:**
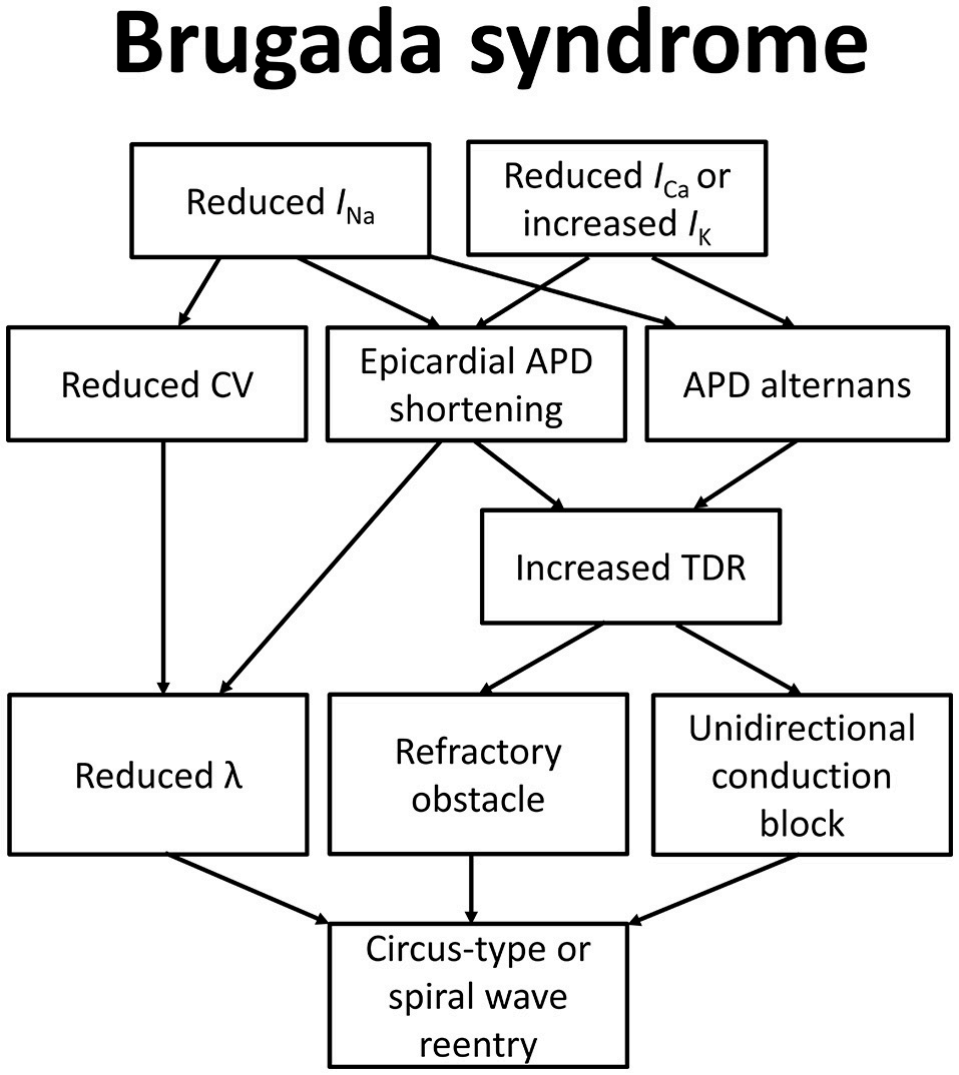
**Molecular and electrophysiological mechanisms underlying arrhythmogenesis in Brugada syndrome**.

To explain how these molecular changes lead to the BrS phenotype, the depolarization, repolarization, and current-to-load mismatch hypotheses have been proposed, as summarized in Figure 1 (Nishii et al., 2010; Wilde et al., 2010; Tse et al., 2016i). Even some 37 years before Martini and colleagues described their syndrome of right bundle branch block, ST segment elevation and sudden death (Nava et al., 1988; Martini et al., 1989), delayed depolarization was thought to underlie ECG changes in a healthy male patient simulating acute myocardial infarction (Osher and Wolff, 1953). Regarding the electrophysiological abnormalities observed in this case, they wrote: “due to prolongation of the depolarization process by right bundle block or possibly focal block with delayed activation of a portion of the right ventricle: unusually early onset of repolarization may also play a role”. Since then, the relative contributions of depolarization vs. repolarization have been intensively studied in pre-clinical models (Antzelevitch et al., 2005, 2007; Antzelevitch, 2008; Schweizer et al., 2014). More recently, a third hypothesis based on electrotonic current posits that current-to-load mismatch in the RV and RVOT subepicardium is responsible for ST segment elevation in BrS (Hoogendijk et al., 2010).

### Depolarization hypothesis

The depolarization theory proposes that slower upstroke of phase 0 and the consequent reduction in conduction velocity (CV) of the APs are responsible for arrhythmogenesis. The seminal work by Martini and colleagues found that patients suffering from RBBB and ST segment elevation also had fibrosis of the right ventricle and the conduction system (Martini et al., 1989). Indeed, targeted disruption of Scn5a (Scn5a^+/−^), Scn5a^1798insD/+^ and SCN5a^G1408R^ mice demonstrate reduced CV associated with interstitial fibrosis (Leoni et al., 2010; Boukens et al., 2013; Schweizer et al., 2014). Scn5a^+/−^ mice also show progressive conduction defects that are suggestive of Lenègre disease (Royer et al., 2005). Na^+^ channels were assumed to be distinct from gap junctions, which are found predominantly at the ends of cardiomyocytes for mediating intercellular conduction. However, they were later shown to co-localize with gap junctions at the intercalated disks (Cohen, 1996). Indeed, the macrostructure connexome has been coined to describe an interacting network of Na^+^ channels, desomosomal components and gap junctions (Rhett and Gourdie, 2012; Rhett et al., 2013; Agullo-Pascual et al., 2014; Veeraraghavan et al., 2014a,b; Veeraraghavan et al., 2015; George et al., 2015). Autopsy findings support the idea that components of the connexome are not independent of each other, by demonstrating increased myocardial fibrosis from collagen deposition and reduced gap junction expression in the RVOT of hearts from BrS patients (Campuzano and Brugada, 2015; Nademanee et al., 2015).

Mouse hearts with plakophilin-2 missense mutations showed a phenotype that is consistent with arrhythmogenic right ventricular dysplasia (ARVD; Cruz et al., 2015). BrS subjects possessing PKP2 mutations have a reduced number of Na^+^ channels at the intercalated disk (Agullo-Pascual et al., 2014; Cerrone and Delmar, 2014; Cerrone et al., 2014; Campuzano et al., 2016). Although ARVD and BrS have been considered to be different disorders, both affect primarily the RV and may represent opposite ends of the same disease spectrum, with decreasing degree of structural abnormalities from ARVD toward BrS (Peters, 2014, 2015). Both ARVD and BrS can be therefore considered as diseases of the connexome.

Together, *Scn5a* models of BrS demonstrate that these conduction disturbances and structural abnormalities are greater in the RV compared to the LV. These are in keeping with clinical findings of delayed depolarization in the RV outflow tract demonstrated using electroanatomical mapping (Nagase et al., 2002; Tukkie et al., 2004; Coronel et al., 2005; Postema et al., 2008, 2010; Lambiase et al., 2009; Nademanee et al., 2011; Ten Sande et al., 2015). Most recently, a panoramic ventricular mapping study in humans showed electrogram prolongation and fractionation, reflecting reduced CV and increased CV dispersion (Zhang et al., 2015). Furthermore, catheter ablation of the RVOT led to resolution of the BrS ECG pattern, and prevention of spontaneous and provoked VT/VF episodes, thereby supporting the depolarization hypothesis (Nademanee et al., 2011; Brugada et al., 2015). However, abnormal depolarization may be less relevant in BrS pathogenic variants in which the *I*_Na_ is not affected. For example, mutations leading to reduced *I*_Ca_ or increased *I*_K_ would not be expected to influence the AP upstroke but rather shortens the plateau phase of AP repolarization.

### Repolarization hypothesis

The repolarization theory states that differential APD shortening across the myocardial wall is primarily responsible for the BrS phenotype. Loss-of-function Scn5a mutations can have opposing effects on the fast and slow inactivation of Na^+^ channels with distinct effects on repolarization (Veldkamp et al., 2000). Evidence in support of the repolarization hypothesis for BrS has been derived from experiments performed in animal models (Di Diego and Antzelevitch, 2003; Antzelevitch and Oliva, 2006; Antzelevitch, 2006; Tsuboi and Antzelevitch, 2006; Sicouri et al., 2012). Data from mouse *Scn5a*^+/−^ hearts on repolarization durations have been inconsistent (Stokoe et al., 2007; Martin et al., 2010, 2011a,b), which has been discussed by editorials elsewhere (Tse et al., 2016f,h,i). Experiments derived from arterially perfused, canine wedge preparations have provided much insights into the mechanisms by which reduced inward currents contributes to heterogeneities in repolarization and in turn reentry (Yan and Antzelevitch, 1999; Antzelevitch, 2001; Fish and Antzelevitch, 2004, 2008). A greater degree of shortening is observed in the epicardium with high *I*_to_ (particularly in the RVOT epicardium) compared to the endocardium with low *I*_to_. Therefore, epicardial APs, but not endocardial APs, lose their dome shape morphology. During phase 1 of the AP, the outward shift of ionic current balance leads to failure of LTCC activation, which also contributes to the loss of the AP dome. Originally, phase 2 reentry was hypothesized to involve electrotonic coupling between these cardiac regions, which permits propagation of the AP dome from endocardial regions where it is maintained to epicardial regions where it is abolished, thereby producing an extrasystole (Yan and Antzelevitch, 1999). Recent work has suggested that such phase 2 reentry is due to heterogeneity in early repolarization in the sub-epicardial layer (Maoz et al., 2014). This can then serve to initiate arrhythmia, and sustain reentry by a circus-type mechanism (Lukas and Antzelevitch, 1996). Steep and reversal of repolarization gradients lead to the ST segment elevation and T-wave inversion, respectively, in the ECG.

However, the above data from wedge preparations on abnormal repolarization must be recognized with some caution, as no significant transmural differences in repolarization durations were found in the clinical setting (Coronel et al., 2005). The ion currents that apparently mediate the propagation of action potential domes across the myocardium can also equilibrate action potential morphology, thereby reducing transmural differences in repolarization time. Nevertheless, increased T_peak_ − T_end_, a marker of global dispersion of repolarization, have been found in BrS patients compared to healthy subjects (Morita et al., 2008; Sangawa et al., 2009; Karim Talib et al., 2012; Maury et al., 2015). Moreover, using non-invasive ECG imaging, abnormal substrates in the form of conduction slowing, delayed epicardial repolarization and increased spatial gradients of repolarization have been identified in the right ventricular outflow tract (Zhang et al., 2015). The findings of this study suggest that abnormal repolarization causes ST segment elevation, whereas abnormal depolarization is responsible for the arrhythmic phenotype observed in BrS (Zhang et al., 2015). For BrS patients in whom *I*_Ca_ is reduced or *I*_K_ is increased, abnormal repolarization is likely to be important in generating or sustaining VT/VF. Indeed, subjects with loss-of-function Ca^2+^ channel mutations have shortened QT intervals, which may support arrhythmias by a reentrant mechanism (Antzelevitch et al., 2007).

Abnormal restitution has long been recognized as a key determinant of arrhythmogenicity in arrhythmic disorders such as long QT and Brugada syndromes (Nishii et al., 2010; Osadchii et al., 2010; Osadchii, 2012a,b, 2013; Tse et al., 2016c,e). Restitution describes the dependence of a parameter on the previous diastolic interval (DI) (Nolasco and Dahlen, 1968; Franz et al., 1983; Tse et al., 2016g). Increased restitution gradients are associated with arrhythmogenesis, likely via the development of APD alternans, which manifest as T-wave alternans on the ECG (Pastore et al., 1999). APD alternans can produce steep gradients in repolarization and refractoriness, unidirectional conduction block and reentry (Tse et al., 2016a).

However, restitution analysis alone underestimates the extent of APD alternans (Matthews et al., 2012) and may not reliably predict arrhythmogenicity (Tse et al., 2016g). This may be due to restitution-independent mechanisms in the generation of alternans (Wu and Patwardhan, 2006; Jing and Patwardhan, 2012), such as divergence of ERP from APD (Tse et al., 2016c,d,e). APD alternans have been demonstrated in a canine model of BrS (Morita et al., 2006). T-wave alternans have also been in Brugada subjects, lending support to the hypothesis that repolarization abnormalities play a role in arrhythmogenesis (Nishizaki et al., 2005; Uchimura-Makita et al., 2014; Sakamoto et al., 2016).

### Current-load-mismatch, depolarization-repolarization balance and excitation wavelength (λ)

A third mechanism involves current-to-load mismatch in the right ventricle (Hoogendijk et al., 2010). This may occur at sites with structural abnormalities, where the myocardial tissue is interspersed with collagen or adipose tissue (Ten Sande et al., 2015). In a preclinical study, ajmaline-mediated sodium channel blockade led to conduction block and excitatory failure, which were associated with ST segment elevation in the pseudo-ECG recordings (Hoogendijk et al., 2011). Computational modeling work demonstrated that the balance between inward and outward currents could affect excitation and the ST segment elevation in concert (Hoogendijk et al., 2011). Thus, either reduced *I*_to_ or increased *I*_Ca_ could compensate for the reduced sodium current, in turn reducing the degree of ST segment elevation. Such preclinical findings are consistent with human data (Hoogendijk et al., 2010). In an explanted human heart, only failure of local excitation, but not delayed activation or early repolarization, correlated with ST segment elevation (Hoogendijk et al., 2010).

In patients with Brugada syndrome, structural abnormalities are indeed observed in the RV and RVOT, which would increase current-load mismatch and excitation failure (Coronel et al., 2005; Frustaci et al., 2005; Catalano et al., 2009). In a recent clinical study, a cohort of BrS patients underwent activation mapping procedures, which showed that electrogram fractionation together with conduction delay and electrocardiographic ST segment elevation were likely due to structural abnormalities in the sub-epicardial region of the right ventricle and right ventricular outflow tract (Ten Sande et al., 2015). It was suggested that current-to-load mismatches at discontinuities can cause conduction block. It should be recognized that abnormal depolarization does not act in isolation, but act in concert with discontinuous conduction to produce arrhythmias in BrS (Postema et al., 2008). This notion is consistent with the observations that BrS patients develop ventricular arrhythmias in their thirties, when interstitial fibrosis is more evident (Coronel et al., 2005; Schweizer et al., 2014; Nademanee et al., 2015). It also interacts with action potential repolarization and recovery to determine the excitation (λ) given by CV × ERP. Decreased λ has been associated with increased likelihood of reentrant arrhythmias not only in pre-clinical animal models, but also in BrS patients (Robyns et al., 2016; Tse, in press; Tse and Yan, in press).

## Concluding remarks

In conclusion, an increasing number of genes have been implicated in the pathogenesis of BrS. The genotype-phenotype correlation for many of these variants are poorly characterized. The commonest gene affected, SCN5A, is only implicated in approximately a fifth of patients. In some pedigrees, affected members did not have mutations in this gene (Probst et al., 2009). On genetic diseases, Marian wrote that “by definition the causal mutation cannot be absent in family members with the phenotype” (Marian, 2009). Therefore, other causal genes remain to be discovered. The use of animal models will be crucial in elucidating the electrophysiological mechanisms of arrhythmogenesis in these cases. Stem cell derived cardiomyocytes can be used to construct monolayers or organoid chambers (Kong et al., 2010; Weng et al., 2014), which can serve as useful platforms for disease modeling, high-throughput drug screening and cardiotoxicity testing (Li, 2012; Lui et al., 2012; Chow et al., 2013). Future studies will be needed to improve risk stratification strategies to determine the subset of patients with Brugada syndrome requiring ICD insertion.

## Author contributions

GT: Design of manuscript; interpreted primary research papers; drafted and critically revised the manuscript for important intellectual content; creation of figure. VL: Interpreted primary research papers. Critically revised the manuscript for important intellectual content. KL: Interpreted primary research papers; drafted and critically revised the manuscript for important intellectual content. VL: Critically revised the manuscript for important intellectual content. YC: Drafted and critically revised the manuscript for important intellectual content. WK: Drafted and critically revised the manuscript for important intellectual content. RL: Critically revised the manuscript for important intellectual content. BY: Drafted and critically revised the manuscript for important intellectual content.

### Conflict of interest statement

The authors declare that the research was conducted in the absence of any commercial or financial relationships that could be construed as a potential conflict of interest.
